# Potential Receptors for Targeted Imaging of Lymph Node Metastases in Penile Cancer

**DOI:** 10.3390/diagnostics10090694

**Published:** 2020-09-15

**Authors:** Christa A. M. van der Fels, Selma Palthe, Henk Buikema, Marius C. van den Heuvel, Annemarie Leliveld, Igle Jan de Jong

**Affiliations:** 1 Department of Urology, University Medical Center Groningen, University of Groningen, 9700 RB Groningen, The Netherlands; selmapalthe@gmail.com (S.P.); a.m.leliveld@umcg.nl (A.L.); i.j.de.jong@umcg.nl (I.J.d.J.); 2 Department of Pathology, University Medical Center Groningen, University of Groningen, 9700 RB Groningen, The Netherlands; h.j.buikema@umcg.nl (H.B.); m.c.van.den.heuvel@umcg.nl (M.C.v.d.H.)

**Keywords:** squamous cell carcinoma, penile neoplasms, lymph nodes, antigens, molecular imaging

## Abstract

Imaging modalities using tumor-directed monoclonal antibodies may be of value to improve the pre- and intraoperative detection and resection of lymph node (LN) metastatic disease in penile squamous cell carcinoma (PSCC). We investigated the expression of prostate-specific membrane antigen (PSMA), vascular endothelial growth factor (VEGF), epidermal growth factor receptor (EGFR) and epithelial cell adhesion molecule (EpCAM) to analyze their potency for diagnostic applications. Antigen expression was determined in primary tumors and LNs with and without metastases of 22 patients with PSCC. The total immunostaining score (TIS, 0–12) was determined as the product of a proportion score (PS, 0–4) and an intensity score (IS, 0–3). EGFR and VEGF expression were high in primary tumor (median TIS 8) and LN metastases (median TIS 6 and 8, respectively). No EGFR expression was seen in LNs without metastases. However, LNs without metastases did show VEGF expression (median TIS 6). No EpCAM or PSMA expression was seen in PSCC. This study shows that VEGF and EGFR expression is moderate to high in LN metastases of PSCC. Both VEGF and EGFR warrant further clinical evaluation to determine their value as a target for pre- and intraoperative imaging modalities in the detection of LN metastases in PSCC.

## 1. Introduction

Early radical resection of lymph node (LN) metastases in patients with penile squamous cell carcinoma (PSCC) is paramount, since this is the only treatment to cure patients with lymph node positive disease [1]. However, radical inguinal LN dissections without LN positive disease are very common, especially in clinically node negative (cN0) patients [2]. LN resection procedures of the groin are invasive with a high risk of complications, including wound infections, skin necrosis, lymphedema and lymphocele formation [3]. Therefore, resection of negative LNs is not desirable. In the last two decades, dynamic sentinel-node biopsy (DSNB) replaced standard LN dissection in the majority of cN0 patients [4]. The DSNB procedure reduces treatment-related morbidity. Still, false-negative nodes in DSNB have been reported [1].

The currently available imaging modalities do not detect small metastases (<10 mm), so they are not useful in staging patients with non-palpable inguinal nodes. New targeted (bio-optical) imaging modalities using tumor-directed monoclonal antibodies may be of value to improve the pre- and intraoperative detection and resection of LN metastatic disease in penile cancer. The monoclonal antibodies prostate-specific membrane antigen (PSMA), vascular endothelial growth factor (VEGF) and epidermal growth factor receptor (EGFR) have already been used as imaging agents for molecular imaging in various tumors. Prostate-specific membrane antigen (PSMA) is highly expressed in prostate tissue and prostate carcinoma. In addition, PSMA expression is also detected in the neovasculature of renal cell carcinoma, transitional cell carcinoma, colon carcinoma and embryonal cell carcinoma [5]. Ga^68^- and F^18^-labeled PSMA tracers have been developed for the use of PET-CT in thee diagnostic evaluation of prostate carcinoma [6]. Vascular endothelial growth factor (VEGF) is overexpressed in a variety of tumors, including gliomas and carcinomas of the breast, kidney, liver and prostate [7]. Squamous cell carcinomas of the head and neck showed a high expression of epidermal growth factor receptor (EGFR) [8]. Both VEGF and EGFR have been used as a radiolabeled imaging agent for molecular imaging in the previously mentioned tumors [9,10]. Previously we reported epithelial cell adhesion molecule (EpCAM) to be an antigen with high tumor distinctiveness for LN positive disease in urothelial cell carcinoma (UCC) of the bladder [11]. Besides imaging modalities, the above mentioned antigens have been used for the development of therapeutic purposes as well.

We hypothesize that one of these antigens could also be a potential protein for the detection of LN positive PSCC in the diagnostic setting. Therefore, we investigated the expression of PSMA, VEGF, EGFR and EpCAM using immunohistochemistry in LN metastatic disease of PSCC. 

## 2. Materials and Methods

### 2.1. Patient Samples

A total of 22 patients with PSCC treated in our hospital were selected as objects of this pilot study. The primary tumors and 25 lymph node metastases of these patients were available for immunohistochemistry. All tissue specimens were anonymously coded. The Medical Ethics Review Board of the University Medical Center Groningen approved this study on 14 December 2017 (METc UMCG 2017/639). Trial registration number (UMCG Research Register): 201700868.

### 2.2. Immunohistochemistry

EpCAM, PSMA and VEGF expression on the primary tumor, LN metastases and tumor-negative LNs were determined by immunohistochemistry (IHC) on 4 micrometer-thick paraffin embedded slides. Normal colon (EpCAM), prostate carcinoma (PSMA) and colon carcinoma (VEGF) served as positive control specimens. Omission of the primary antibody on positive control specimens served as negative controls.

Slides were deparaffinized with xylene baths and decreasing grades of alcohol. Depending on the primary antibody, different methods of antigen retrieval were used. For EpCAM incubation, antigen retrieval with 0.1% protease for 30 min at room temperature was used. For PSMA and VEGF, antigen retrieval was performed by heating microwave (500 W) for 15 min in a 10 mM citrate buffer at pH 6.0, with a cool down period for 15 min afterwards. Endogeneous peroxidase was blocked with 0.3% hydrogen peroxide in PBS for 20 min in the dark. Slides were incubated with the primary antibodies, diluted in 1% BSA/PBS for 1 h at room temperature with mouse monoclonal antibody (AB) anti-EpCAM (1:100, clone VU-1D9, Leica Biosystems, Newcastle, UK), mouse monoclonal AB anti-PSMA (1:50, YPSMA-1, clone sc-59674, Santa Cruz Biotechnology, Santa Cruz, CA, USA), and mouse monoclonal AB anti-VEGF (C-1) (1:100, clone sc-7269, Santa Cruz Biotechnology, Santa Cruz, CA, USA). In the secondary step, slides were incubated with rabbit anti-mouse AB conjugated to polymer-horseradish peroxidase (DAKO, Glostrup, Denmark), diluted at 1:100 in 1% BSA/PBS with 1% AB serum. In the tertiary step goat anti-rabbit AB conjugated to polymer-horseradish peroxidase (DAKO, Glostrup, Denmark) was used, diluted at 1:100 in 1% BSA/PBS with 1% AB serum. Secondary and tertiary antibodies were incubated for 30 min at room temperature. After every step, slides were washed with PBS and dried. Next, slides were immersed for 10 min in a solution of 0.05% 3, 3′-diaminobenzidine (Sigma-Aldrich, Steinheim, Germany) and 0.03% hydrogen peroxide in PBS in the dark for visualization of the signal as brown staining. After washing with demineralized water, slides were slightly counterstained with haematoxylin, dehydrated by increasing grades of alcohol and when dried, mounted with Tissue Tek film (Sakura Finetek, Leiden, The Netherlands).

Staining of the antigens was compared to the staining of Pan-Cytokeratin (CK AE1/AE3), an epithelial marker that is positive in the vast majority of PSCC, to secure the accuracy and reliability of our project.

For CK AE1/AE3 and EGFR, immunohistochemistry was performed in the Autostainer Link 48 (Dako). This includes antigen retrieval (4 min) in protease 3, incubation with the primary antibody (16 min for Anti-CK AE1/AE3; 8 min for Anti-EGFR) and incubation with the visualization complex (8 min). Counterstaining was performed with haematoxylin.

### 2.3. Evaluation of Staining

The immunostaining was evaluated semi quantitatively by an experienced genitourinary pathologist (M.H.), in a manner that was described previously [11,12,13]. The proportion score (PS) multiplied by the intensity score (IS) resulted in the total immunostaining (TIS). The PS, representing the proportion of the tumor positive for an antigen staining, was scored as 0, none; 1, <10%; 2, 10–50%; 3, 51–80%; 4, >80. The IS, representing the intensity of immunostaining, was scored as 0, no staining; 1, weak; 2, moderate; 3, strong. As IS scoring may be subject to intraobserver variability, IS was also scored digitally using the Visiopharm intensity app. No significant differences in IS scoring when comparing the scores of M.H. and the app were present. The IS scores generated by the Visiopharm app were used in this study.

### 2.4. Data Analysis

Descriptive analyses were used to describe the results and are shown as median values with interquartile range. SPSS statistics (version 23.0 for Windows, IBM Corp, Armonk, NY, USA) was used for analyses.

## 3. Results

**Figure 1:**
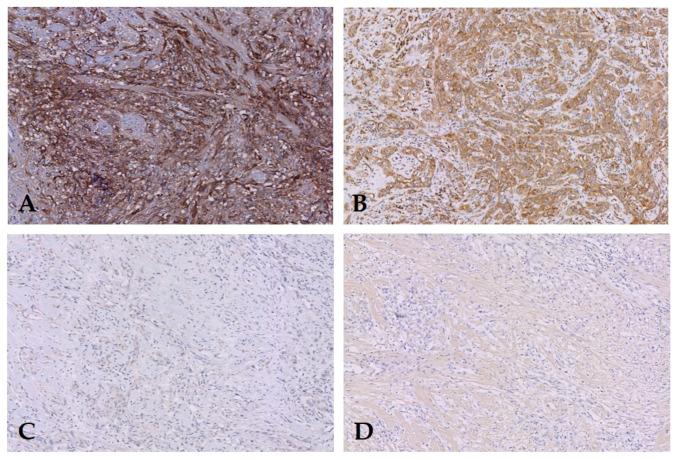
Primary tumor of one patient with (**A**) EGFR and (**B**) VEGF expression but absence of (**C**) prostate-specific membrane antigen (PSMA) and (**D)** EpCAM expression. Original magnification 200×.

**Figure 2:**
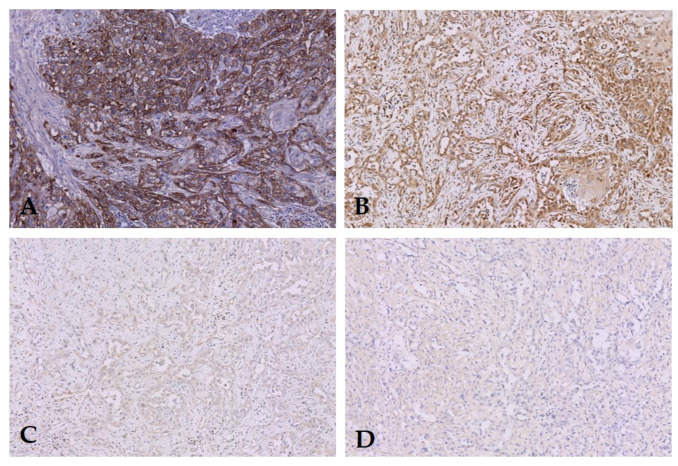
Lymph node metastasis of one patient with expression of (**A**) EGFR; (**B**) VEGF; no expression of (**C**) PSMA and (**D**) EpCAM. Original magnification 200×.

**Figure 3:**
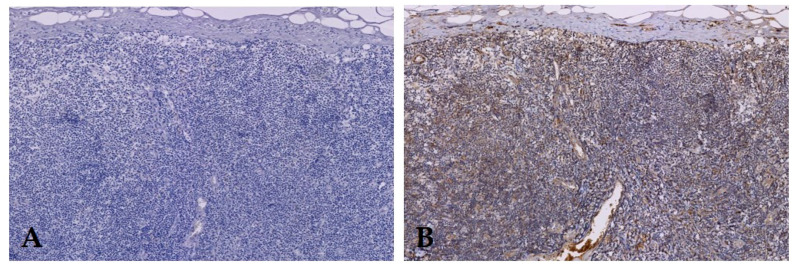
Lymph node without metastasis without (**A**) EGFR expression, but with (**B**) VEGF expression. Original magnification 200×.

The proportion score of VEGF in both primary tumors (median proportion score (PS) 4) and metastatic LNs (median PS 4) of PSCC was high (Table 1). The intensity score was more variable (with a median intensity score (IS) of 2 in primary tumors and in metastatic LNs). This resulted in a high VEGF expression in primary tumors and in metastatic LNs (median total immunostaining score (TIS) 8, Figure 1 and Figure 2). In addition, VEGF expression was also present in lymphoid tissue of LNs without metastasis (median TIS 6). Figure 3. This resulted in a ratio between tumor-to-no tumor tissue of 1.3 ± 1.3 (median ± SD). 

EGFR expression was prominent in both primary tumors (median TIS 8, Figure 1) and LN metastases (median TIS 6, Figure 2). Primary tumors as well as LN metastases showed a median proportion score of 3. Median intensity score in primary tumor was 2.5 (IQR 2–3) and in LN metastases 2 (IQR 1–2) Table 1. Expression of EGFR was absent in LNs without metastases (Figure 3). In order to test expression of PSMA and EpCAM in PSCC, a pilot study using five patients was performed (Table 2). No EpCAM expression was seen in PSCC at all. PSMA expression was only present in endothelial cells surrounding tumor tissue; the primary tumors were negative (Figure 1 and Figure 2). Therefore, no further samples were tested for PSMA and EpCAM expression. 

**Figure 4:**
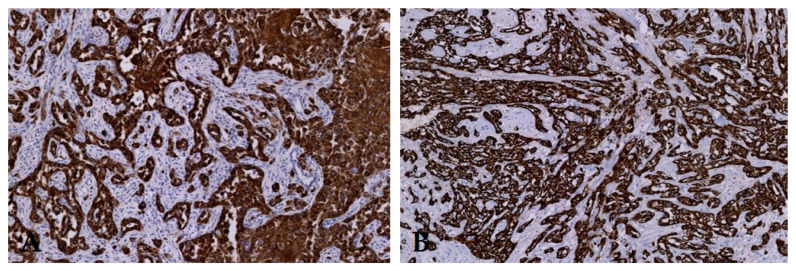
(**A**) Metastasis in a lymph node and (**B**) primary tumor of the same patient with CK AE1/AE3 expression. Original magnification 200×.

As a control for epithelial origin of the tumors, CK AE1/AE3 expression was highly seen in all samples (primary and LN metastases) with PSCC (Figure 4). Median TIS in primary tumors and metastatic LNs was 12 (maximum score) (Table 2). Lymph nodes without PSCC were not tested because no expression was seen in tissue without tumors.

## 4. Discussion

To our knowledge, this is the first study that investigated the expression of different antigens in LN metastatic disease of PSCC. We have shown that VEGF and EGFR are expressed in both primary PSCC and LN metastases. The expression of VEGF in LN positive disease was higher than the expression of EGFR. However, background expression of VEGF in LNs without metastases was also presented in contrast to the expression of EGFR. Different study protocols with different antigen solutions were used, but staining of VEGF in negative LNs remained. PSMA and EpCAM appeared not to be usable as imaging targets for PSCC because the negative staining results of these antigens in primary tumors as well as positive LNs.

We compared our results to the available literature. The immunohistochemical expression of EGFR in primary PSCCs was evaluated by Lavens et al. and showed a positive staining in 100% of the evaluated cases, with strong overexpression in most cases (14/17) [14]. Chaux et al. found in 78.6% of the evaluated cases a positive staining for EGFR [15]. Li et al. found a 53.7% overexpression of VEGF-A in primary PSCCs [16]. No data about expression of these antigens in positive LNs are available. We evaluated the expression of EpCAM and PSMA on PSCC because of the availability of imaging tracers using antibodies against these antigens. Similarly to our results, Froehner et al. also described weak immunohistochemical staining of PSMA in endothelial cells surrounding a positive LN, but the absence of staining in tumor tissue [17]. Chang et al. found PSMA expression in the neovasculature of urologic and a variety of other malignant neoplasms [5]. EpCAM overexpression is associated with advanced stage, high grade and LN metastasis in some tumors [18]. However, no data about immunohistochemical expression of EpCAM on PSCC are available. Other alternative biomarkers for PSCC that have been identified in the literature include p53, with a reported overexpression variation between 26% and 91%, and HPV-related markers such as P16INK4a [19]. However, no imaging modalities with these biomarkers are available yet.

The expression of VEGF and EGFR in PSCC and LN metastases of PSCC could be explained by their properties. VEGF is a protein that is overexpressed in various tumors and is associated with angiogenesis. Angiogenesis plays a key role in tumor development and metastasis [20]. Increased expression of VEGF has been described to be associated with disease progression in renal cell carcinoma [21] and prostate cancer [22]. However, a correlation between VEGF expression and prognosis of PSCC has not been confirmed yet [16]. EGFR is a protein involved in the pathogenesis and progression of different carcinoma types [23]. EGFR overexpression in squamous cell carcinoma in the head and neck areas is known to be associated with a poor prognosis. Moreover, an association between EGFR overexpression and poor prognosis of penile cancer has been described, especially with disease recurrence and reduced survival [24]. On the other hand, other studies could not find an association between EGFR expression and histologic subtype/histologic grade [15]. Unfortunately, because of the low number of patients in this study, we were not able to correlate our staining findings with the tumor characteristics of our patients.

Recently, different studies have shown that anti-EGFR therapy with cetuximab might be useful in the treatment of advanced PSCC [25,26,27] Cetuximab has been administered alone or in combination with chemotherapy to patients with advanced disease. Partial response was seen in lymph node metastatic disease, especially in patients without visceral or bone metastases [28]. More clinical studies are needed to investigate whether anti-EGFR therapy can play a role in the treatment of advanced penile cancer. Anti-VEGF therapy has been used as a cancer treatment in various malignancies, including renal cell cancer and prostate cancer [29], but also in squamous cell carcinomas. As far as we know, no data about anti-VEGF therapy against PSCC are available.

Molecular imaging modalities use the expression of specific antigens of tumor tissue. Near-infrared fluorescent dyes have been coupled to monoclonal antibodies cetuximab and bevacizumab, directed against EGFR and VEGF, respectively, in order to detect tumor tissue [30]. Although tumor EGFR expression is associated with fluorescence intensity, only low levels of EGFR expression are required for imaging [31]. High background uptake in surrounding tissue, for example in muscle, skin or stroma (tissue without tumor) reduces the specificity of these fluorescence-guided optical imaging modalities [32]. This also applies for the expression of antigens in other carcinomas located near the penis. EGFR is overexpressed in prostate cancer, in 31–48% of bladder cancer and 25–77% of colon and rectal cancer as well [33], which might reduce the specificity for the detection of PSCC by using imaging modalities with antibodies directed against EGFR. VEGF is also overexpressed in prostate cancer [7]. The use of PET imaging with antibodies against VEGF in patients with other tumors also showed normal organ uptake of VEGF, including uptake in normal lymph nodes [34], which is in line with our results. Heterogeneous antibody distribution of VEGF within patients has been seen as well as within individual tumors.

Our results show that EpCAM and PSMA are not suitable to use for the detection of PSCC in the diagnostic setting. Based on our study, EGFR seems a more suitable candidate for molecular imaging in PSCC based on the higher tumor-to-background ratio compared to VEGF.

## 5. Conclusions

In this study we demonstrate that both primary PSCC and LN metastases show moderate to high expression of VEGF and EGFR. Both proteins could be candidates as targets for pre- and intraoperative imaging modalities in the detection of LN metastases of PSCC. Due to staining of normal lymphoid tissue in lymph nodes for VEGF, EGFR seems to be the best candidate. Further clinical evaluation is needed to determine their value as a target for pre- and intraoperative imaging modalities in the detection of LN metastases of PSCC.

## Figures and Tables

**Table 1:** Scoring immunoreactivity of vascular endothelial growth factor (VEGF) and epidermal growth factor receptor (EGFR) on penile squamous cell carcinoma (PSCC), median (interquartile range)**.**

		VEGF			EGFR		
	*n*	PS (IQR)	IS (IQR)	TIS (IQR)	PS (IQR)	IS (IQR)	TIS (IQR)
**Primary PSCC**	22	4 (3–4)	2 (2–3)	8 (6–12)	3 (2.75–4)	2.5 (2–3)	8 (6–9.75)
**LN + PSCC**	25	4 (3–4)	2 (2–2)	8 (6–8)	3 (2–3)	2 (1–2)	6 (2–8)
**LN**	22	3 (2–3)	2 (1.75–2)	6 (3.75–6)	0 (0–0)	0 (0–0)	0 (0–0)

PS: proportion score, IS: intensity score, TIS: total immunostaining score, IQR: interquartile range.

**Table 2:** Scoring immunoreactivity of all antibodies on PSCC, median (interquartile range)**.**

	VEGF		EGFR		PSMA		EpCAM		CK AE1/AE3	
	*n*	TIS (IQR)	*n*	TIS (IQR)	*n*	TIS (IQR)	*n*	TIS (IQR)	*n*	TIS (IQR)
**Primary PSCC**	22	8 (6–12)	22	8 (6–10)	5	0 (0–2)	5	0 (0–0)	22	12 (12–12)
**LN + PSCC**	25	8 (6–8)	25	6 (2–8)	5	2 (1–2)	5	0 (0–0)	25	12 (12–12)
**LN**	22	6 (4–6)	22	0 (0–0)						

TIS: Total Immunostaining Score, IQR: interquartile range.

## Abbreviations

AB	Antibody
CK	Cytokeratin
EGFR	Epidermal Growth Factor Receptor
EpCAM	Epithelial Cell Adhesion Molecule
IS	Intensity Score
LN	Lymph Node
PS	Proportion Score
PSCC	Penile Squamous Cell Carcinoma
PSMA	Prostate Specific Membrane Antigen
TIS	Total Immunostaining
VEGF	Vascular Endothelial Growth Factor
